# Supplementary figures and images for: Increased force and elastic energy storage are not the mechanisms that improve jump performance with accentuated eccentric loading during a constrained vertical jump
Source: PLoS One. 2024 Aug 6;19(8):e0308226. doi: 10.1371/journal.pone.0308226 (PMC11302863; doi:10.1371/journal.pone.0308226)

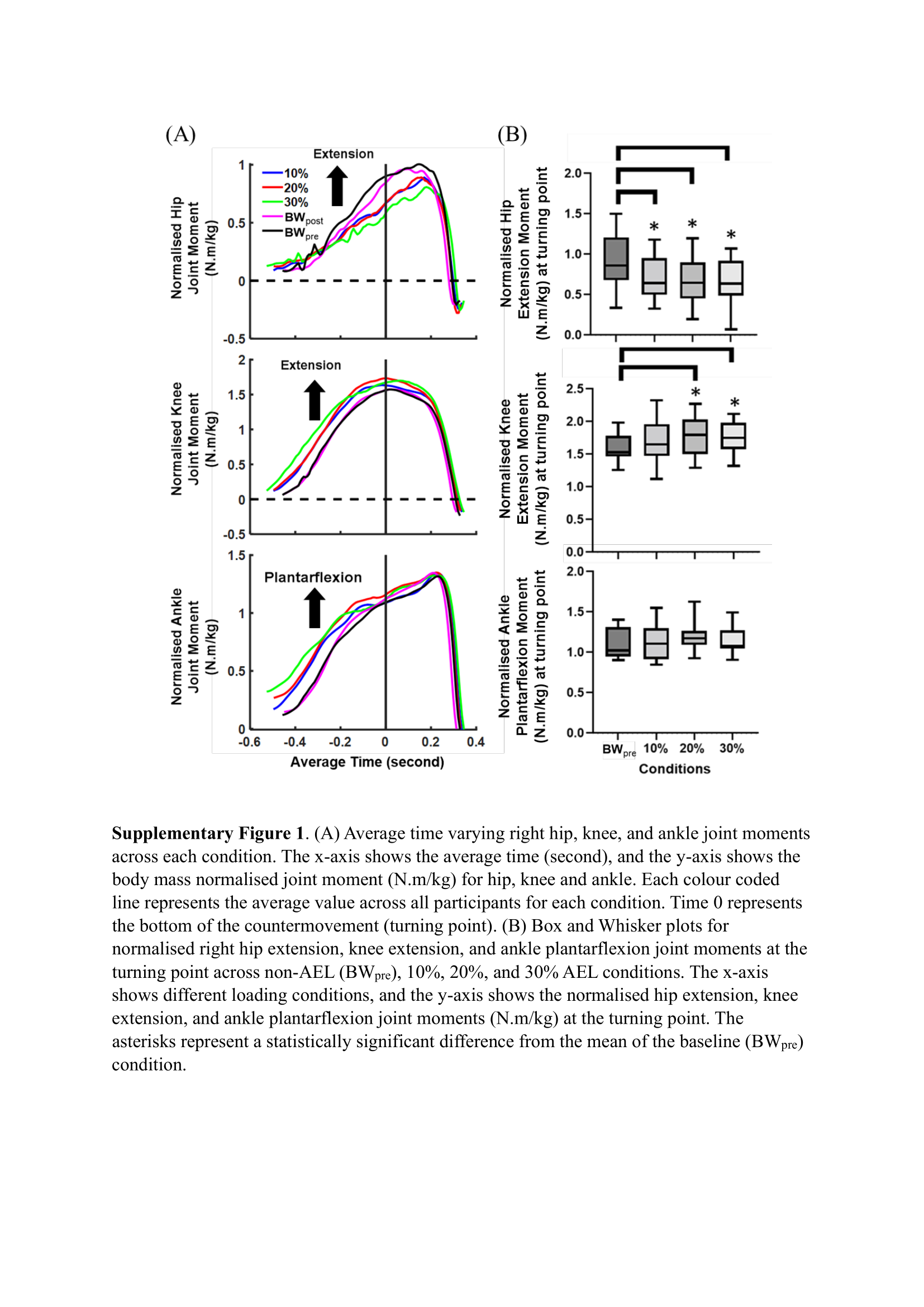

Supplement: S1 Fig — (A) Average time varying right hip, knee, and ankle joint moments across each condition. The x-axis shows the average time (second), and the y-axis shows the body mass normalised joint moment (N.m/kg) for hip, knee and ankle. Each colour coded line represents the average value across all participants for each condition. Time 0 represents the bottom of the countermovement (turning point). (B) Box and Whisker plots for normalised right hip extension, knee extension, and ankle plantarflexion joint moments at the turning point across non-AEL (BWpre), 10%, 20%, and 30% AEL conditions. The x-axis shows different loading conditions, and the y-axis shows the normalised hip extension, knee extension, and ankle plantarflexion joint moments (N.m/kg) at the turning point. The asterisks represent a statistically significant difference from the mean of the baseline (BWpre) condition. (TIF) [file pone.0308226.s001.tif]

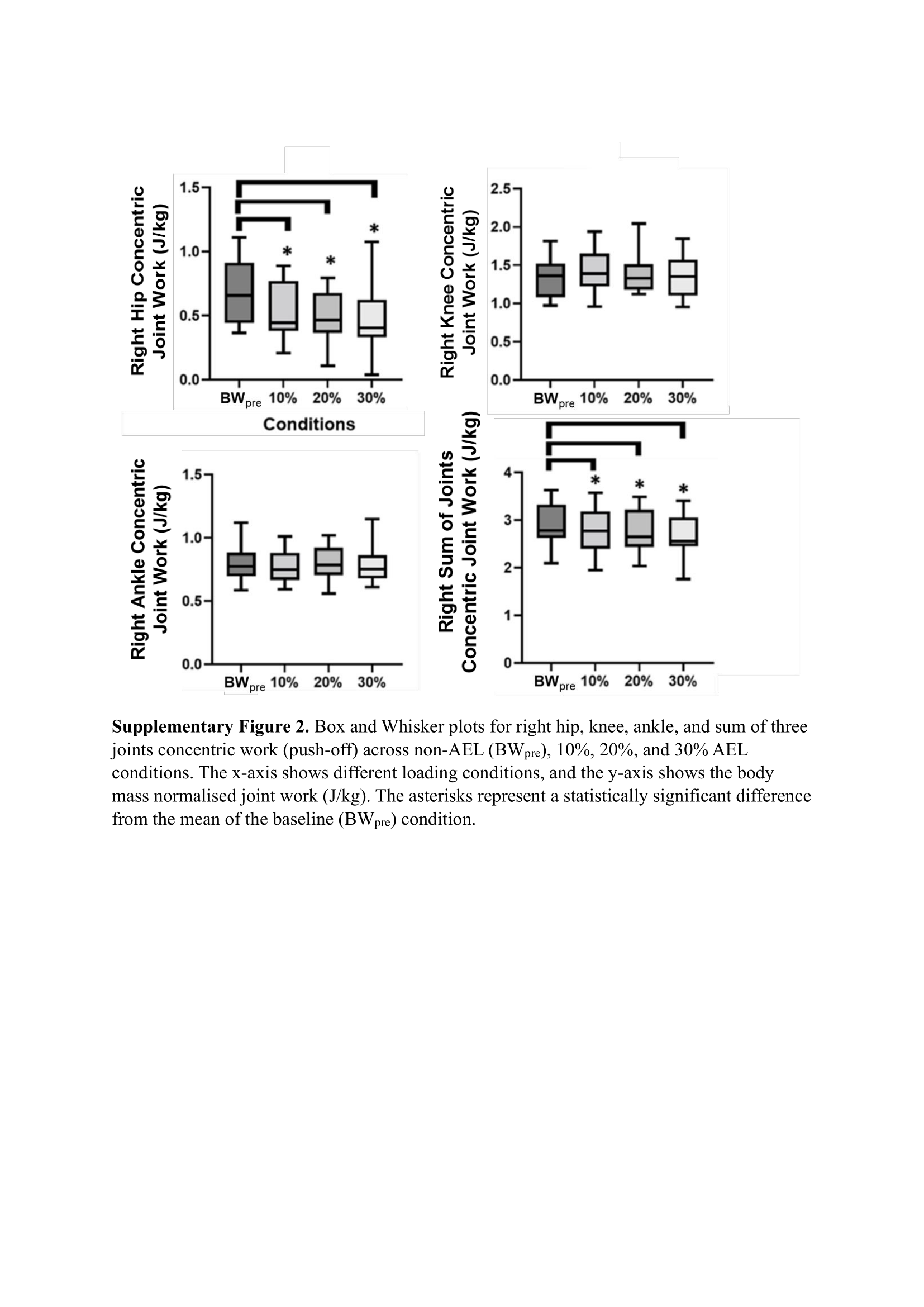

Supplement: S2 Fig — Box and Whisker plots for right hip, knee, ankle, and sum of three joints concentric work (push-off) across non-AEL (BWpre), 10%, 20%, and 30% AEL conditions. The x-axis shows different loading conditions, and the y-axis shows the body mass normalised joint work (J/kg). The asterisks represent a statistically significant difference from the mean of the baseline (BWpre) condition. (TIF) [file pone.0308226.s002.tif]

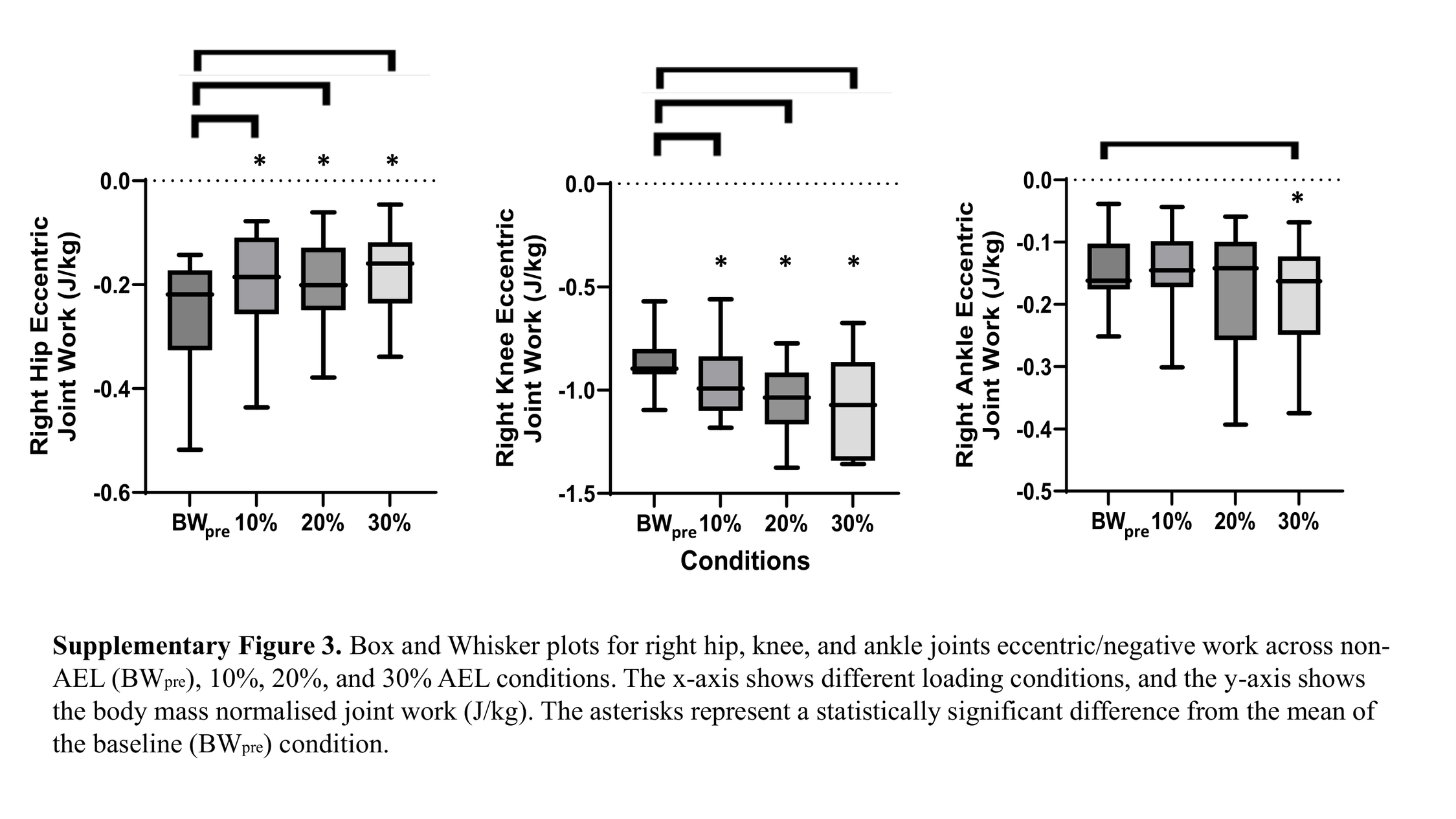

Supplement: S3 Fig — Box and Whisker plots for right hip, knee, and ankle joints eccentric/negative work across non-AEL (BWpre), 10%, 20%, and 30% AEL conditions. The x-axis shows different loading conditions, and the y-axis shows the body mass normalised joint work (J/kg). The asterisks represent a statistically significant difference from the mean of the baseline (BWpre) condition. (TIF) [file pone.0308226.s003.tif]

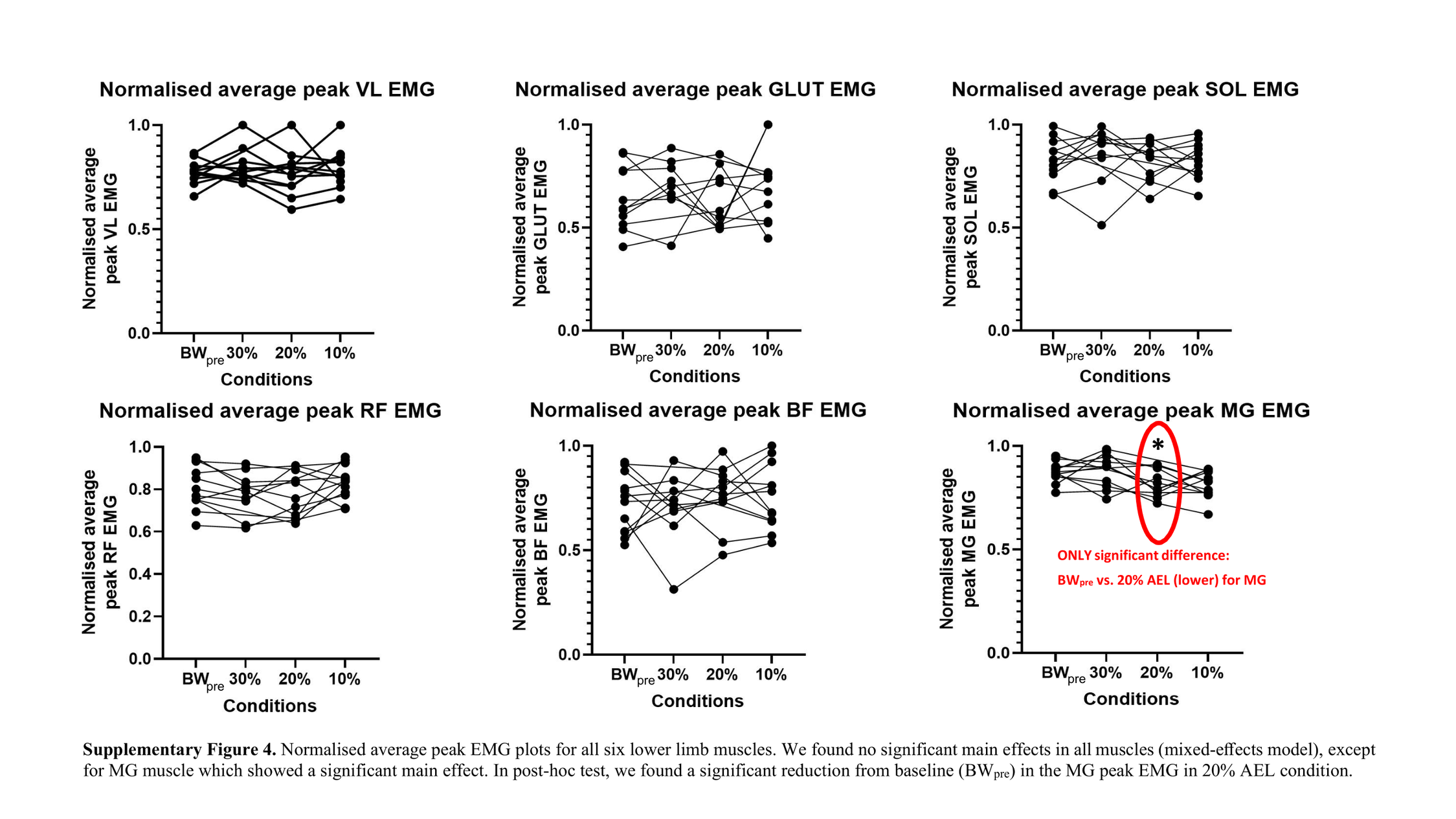

Supplement: S4 Fig — The x-axis shows different loading conditions, and the y-axis shows the normalised average peak EMG. We found no significant main effects in all muscles (mixed-effects model), except for MG muscle which showed a significant main effect. In post-hoc test, we found a significant reduction from baseline (BWpre) in the MG peak EMG in 20% AEL condition. (TIF) [file pone.0308226.s004.tif]

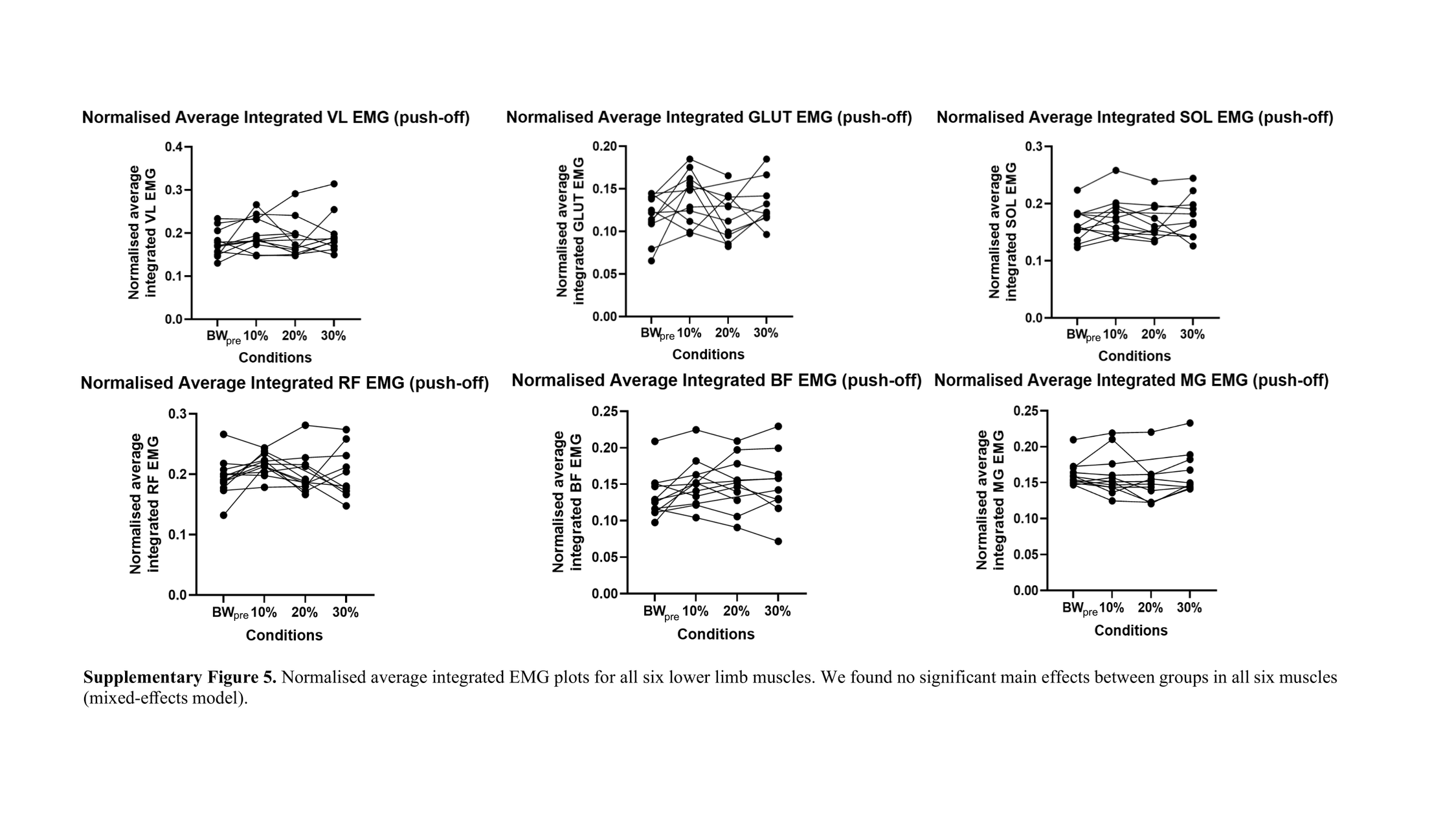

Supplement: S5 Fig — The x-axis shows different loading conditions, and the y-axis shows the normalised average integrated EMG. We found no significant main effects between groups in all six muscles (mixed-effects model). (TIF) [file pone.0308226.s005.tif]

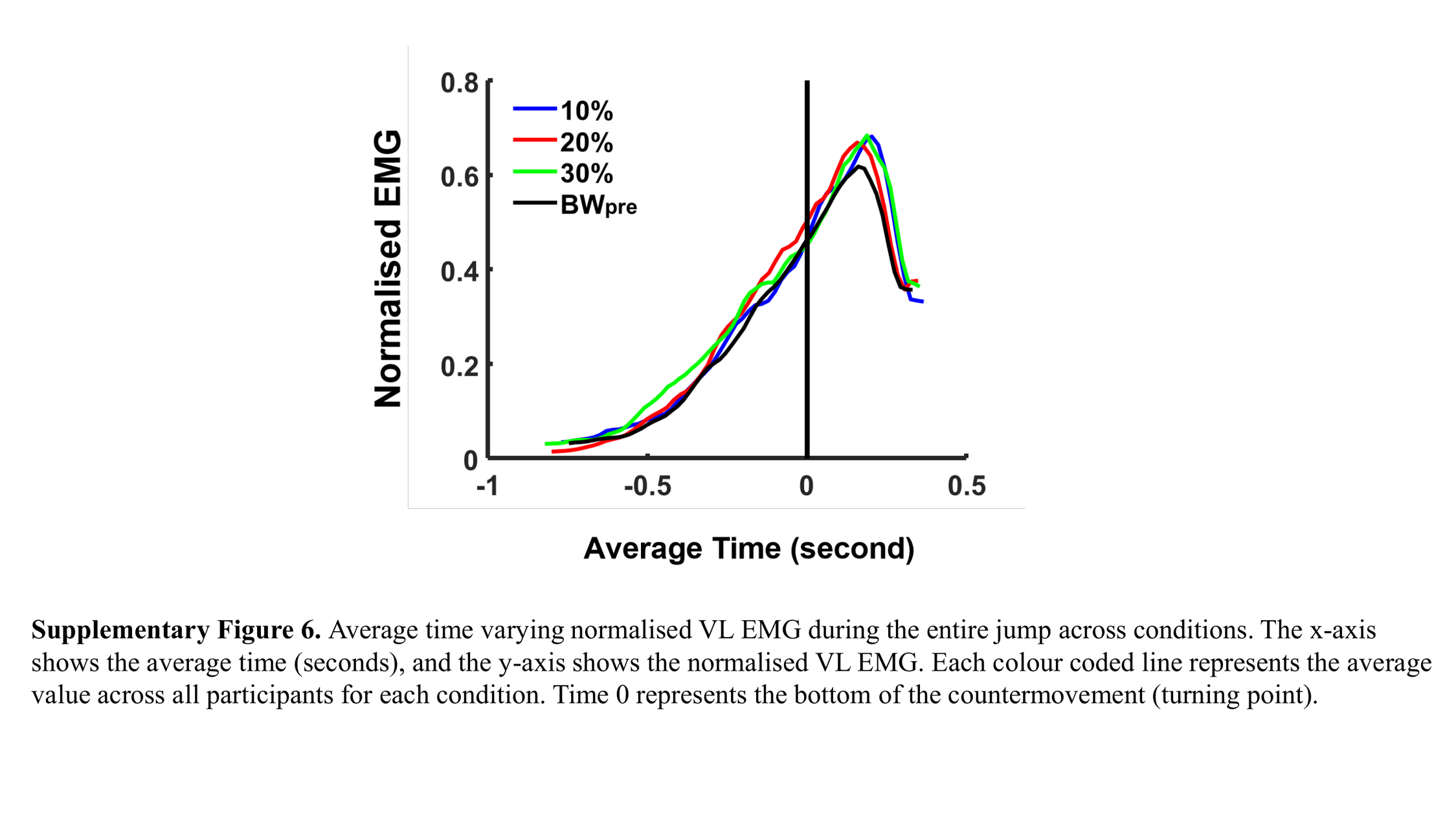

Supplement: S6 Fig — The x-axis shows the average time (seconds), and the y-axis shows the normalised VL EMG. Each colour coded line represents the average value across all participants for each condition. Time 0 represents the bottom of the countermovement (turning point). (TIF) [file pone.0308226.s006.tif]
