# Supplementary material for: Increased force and elastic energy storage are not the mechanisms that improve jump performance with accentuated eccentric loading during a constrained vertical jump
Source: PLoS One. 2024 Aug 6;19(8):e0308226. doi: 10.1371/journal.pone.0308226 (PMC11302863; doi:10.1371/journal.pone.0308226)
Supplement: S1 File — (ZIP) [file pone.0308226.s007.zip › Code/Explanation of MATLAB codes used in this study.docx]

**Explanation of MATLAB codes used in this study**

**Prerequisites of data collection**

This study required data collection of 3D motion capture marker position, EMG, and force plate data in the Qualisys software (Qualisys Track Manager, or QTM). After data collection, users exported the [marker position and force plate data] in C3D files and exported the [EMG data] in MAT files. Data export was completed in QTM software, and no code was needed. The outputs were **C3D** and **MAT** files, and each trial included two of these files.

**C3D files (marker and force plate data)**

We used OpenSim platform to perform all the steps for the calculation of kinematics and kinetics data [model scaling - inverse kinematics - inverse dynamics - body analysis]. Since the process was highly iterative in nature, we used MATLAB scripting option to perform these calculations semi-automatically to avoid relying on OpenSim GUI. To use these codes, users need to download a publicly available toolbox which contains all the MATLAB-OpenSim functions needed to run OpenSim through MATLAB interface. This toolbox is known as “Matlab and the Biomechanical ToolKit (btk)” (https://biomechanical toolkit.github.io/docs/Wrapping/Matlab/_tutorial.html).

Inside the **“Code\Kinematic_kinetic_code”** folder, there is another subfolder called **“opensim_batch_uq_v0_1”,** which is the main processing pipeline for our kinematic and kinetic analysis. In brief, click on **“opensim_batch_uq_v0_1.fig”**. This is the main processing GUI to prompt users to select multiple inputs and the level of analysis required, which then outputs a scaled subject-specific model and the analysis results (i.e., inverse kinematics, inverse dynamics, body analysis). In this study, the default model OSIM file was **“LaiArnold2017_refined_scaled.osim”** (inside the folder **“OpenSim model and setup files”**). We used this default model file to scale all participants’ data. There were four other setup files required for scaling, inverse kinematics (IK), inverse dynamics (ID), and external load file (this was required to align force plate data with OpenSim’s kinematic data). These setup files were required to successfully run IK/ID analysis in this pipeline. For each participant, we collected a static calibration trial (C3D file), and we also selected multiple dynamic C3D files (all the best jumping trials in this study for each participant). These static and dynamic C3D files were the inputs for this pipeline (**“opensim_batch_uq_v0_1.fig”**).

We used various MATLAB codes to perform some smaller data analysis using the results from OpenSim. Inside the folder **“Code\Kinematic_kinetic_code”**, there is a subfolder named **“Analysis and plotting codes”**. All the relevant codes were provided here:

- **“calculate_yCOM_height.m”**: This code calculated the effective jump height as the difference between the highest vertical position of the manubrium marker (MAN) for each trial and the average MAN vertical position in the static trial for each participant. By changing the data access column number inside this code, users could also calculate jump height using the whole model’s COM (instead of MAN marker position).
- **“get_COM_depth.m”**: This code calculated the squat depth, which as the difference between the average MAN vertical position in the static trial for each participant and the lowest MAN vertical position for each trial. Similarly, by changing the data access column number inside this code, users could also calculate squat depth using the whole model’s COM (instead of MAN marker position).
- All data analysis and plotting codes within the **“Analysis and plotting codes”** folder followed the same definition of turning point, take-off frame, and push-off phase according to the manuscript. For example, all codes that required identifying these key frames would include similar code section as below:

% find lowest point time & frame

MAN = ss.(fields{i}).marker_data.Markers.MAN(:,3);

[turn,~]=find(MAN==min(MAN)); turn = turn(1);

turn_time = time(turn); % turning time of the marker

% find take-off time & frame

FP9_1 = ss.(fields{i}).fp_data.GRF_data(1).F(:,3);

FP9_2 = ss.(fields{i}).fp_data.GRF_data(2).F(:,3);

FP9 = FP9_1 + FP9_2;

time_highframe = ss.(fields{i}).fp_data.Time;

[tof,~]=find(FP9<=5); % take-off frame of the GRF

tof_time = time_highframe(tof);

[a,~] = find(tof_time>=turn_time); a = a(1);

tof = tof(a); % first frame after the lowest MAN point time

- Within the folder named **“Re-do-ECC_VGRF”**, there were five codes named **“BW.m”**, **“BWP.m”**, **“Ten.m”**, **“Twenty.m”**, **“Thirty.m”**. These codes searched for the start of the jump, and output VGRF, time, and maximal descent velocity data for each trial. The code first prompted users to use MATLAB Ginput to manually select a baseline range as the initial standing phase. We ensured that for our baseline phase selection, the force fluctuation from VGRF was no larger than 5 N (this was a manual process). Once the selection was completed, the code searched for the instant when the VGRF dropped by more than the biggest force fluctuation in the initial standing phase. In practice, this was the instant when the VGRF decreased by more than 5 N from the initial standing phase for each trial. These codes then output VGRF, time, and maximal descent velocity data, and we stored data as **“Data_Full_Ecc.mat”.** We provided GraphPad file for **“Maximal_Descent_Speed.prism”**. We also used **“plot_full_ecc_VGRF.m”** to plot the VGRF graph with the full eccentric phase. We used the code **“calculate_VGRF_at_turning_point.m”** to obtain the VGRF value the turning point.
- Inside the **“Analysis and plotting codes”**, we used **“plot_avg_time_norm_hip_moment.m”**, **“plot_avg_time_norm_knee_moment.m”**, and **“plot_avg_time_norm_ankle_moment.m”**  to plot average time varying normalised joint moment data. These codes also outputted the joint moment values at the turning point.
- Similarly, inside the **“Analysis and plotting codes”**, we used **“plot_avg_time_norm_COM_power.m” “plot_avg_time_norm_hip_power.m”**, **“plot_avg_time_norm_knee_power.m”**, and **“plot_avg_time_norm_ankle_power.m”**  to plot average time varying normalised COM and joint power data. These codes also outputted the peak positive COM and joint power data.
- We used **“calculate_normalized_across_COM_push_off_work.m”**, **“calculate_normalized_across_hip_push_off_work.m”**, **“calculate_normalized_across_knee_push_off_work.m”**, and **“calculate_normalized_across_ankle_push_off_work.m”** to calculate concentric joint and vertical COM work during the push-off phase. We then manually summed the concentric joint work across hip, knee, and ankle joints to calculate the sum of lower limb joints concentric work. Using the same codes, we adjusted the integration frames inside the code (manual process) so that eccentric joint work was calculated by integrating the negative joint power during the eccentric phase (descent).

**MAT files (EMG processing and analysis)**

Folder named **“Code\EMG_code”** contains all codes required to analyse EMG data for this study. Raw EMG data (MAT files) were collected at 1250 Hz and were directly accessible in MATLAB. As explained in the manuscript, raw EMG data were zero-phase band-pass filtered (30-350 Hz second-order Butterworth filter), rectified, and zero-phase low-pass filtered (5 Hz second-order Butterworth filter) to form a linear envelop as the “processed EMG data.” We plotted the trial-by-trial processed EMG linear envelop over time (for each participant, per muscle) using the MATLAB code **“EMG_analyzing.m”**. The key MATLAB code section to perform this EMG processing is as follow:

d = designfilt('bandpassiir','FilterOrder',2, ...

'HalfPowerFrequency1',30,'HalfPowerFrequency2',350, ...

'SampleRate',1250);

**“EMG_analyzing.m”** allowed users to select the EMG Channel of interest (i.e., BF, which stands for biceps femoris) to plot the EMG linear envelops for the same participant and same muscle across different trials per condition. Users needed to manually type in different EMG Channel of interest (i.e., VL, GLUT, etc.) as shown in the code section below:

BF_raw = matfile.analog_data.Channels.BF; (changed “BF” to “VL”, for example)

Users then examined the linear envelop plots to exclude trials where data drop-out occurred due to technical error AND this drop-out prevented further analysis (this step involved manual identification).

From there, we used the MATLAB code **“selected_VL_EMG_peak_value_time.m”** to determine the raw VL peak EMG value for all eligible trials. We then used the code “**selected_VL_integrated_EMG.m”** to determine the raw VL integrated EMG during the push-off phase. We performed the same analysis with the rest of the muscles (SOL, MG, GLUT, BF, RF), and the code naming system was according to these muscle abbreviations (these MATLAB codes are provided within the folder **“Code\EMG_code”**).

EMG data were expressed as absolute values, and we copied and pasted these values into an excel spreadsheet called **“selected_EMG_peak_value_time.xlsx”** to record the individual trial’s absolute peak EMG, and into another excel spreadsheet called **“selected_integrated_EMG.xlsx”** to record the individual trial’s absolute integrated EMG during push-off (see folder **“EMG_code\EMG data”**). We perform EMG normalisation for each participant per muscle in these excel sheets. First, for the same participant (i.e., P01) and the same muscle (i.e., VL), we selected the highest absolute peak EMG value as the maximum reference for that muscle for each participant. We then calculated the absolute average peak EMG for each participant per muscle and per condition, and normalise the value to the maximum reference for that muscle (see excel spreadsheet **“selected_EMG_peak_value_time.xlsx”** for details). Similarly, we then used the same maximum reference value to normalise the average integrated EMG in **“selected_integrated_EMG.xlsx”**. We further stored these same EMG processed data into **“average_peak_EMG.xlsx”** and **“integrated_EMG.xlsx”** and used plotting codes named **“average_peak_EMG_plotting.m”** and **“integrated_EMG_plotting.m”** to plot all our EMG scatter plots in our manuscript (see folder **“Code\EMG_Code”**). We also used the code **“get_VL_emg_envelop.m”** to generate S6_Fig; however, this code required extensive manual trial identification process. We include our GraphPad Prism files (statistical results) for your reference: **“selected_average_peak_EMG.pzfx”** and **” selected_integrated_EMG.pzfx”**. (see folder **“EMG_Code\EMG data”**)
