# Supplementary material for: Increased force and elastic energy storage are not the mechanisms that improve jump performance with accentuated eccentric loading during a constrained vertical jump
Source: PLoS One. 2024 Aug 6;19(8):e0308226. doi: 10.1371/journal.pone.0308226 (PMC11302863; doi:10.1371/journal.pone.0308226)
Supplement: Attachment — Submitted filename: Response to Reviewers.docx [file pone.0308226.s008.docx]

**Note: line numbers in our responses are based on the clean manuscript (not track changes).**

Reviewer #1: General Comments:
This study was to investigate the effect of additional eccentric loading on jump performance (jumping height and power). It was a very interesting topic and the entire study was well executed. In general, if the load in the ECC phase is higher (i.e., higher activity level), the jump height should increase because the load is lighter in the CON phase (since the additional load is removed in the CON phase in this study). Previous studies showed that optimal loading of the ECC phase existed with squat exercise (Takarada et al. 1997), drop-jump (Komi and Bosco 1978). However, in the present study, the work of hip joint and center of mass (and jumping height) decreased under high eccentric loading condition. This result may be related to the fact that the subjects were not giving their all due to the sudden load removal when switching from the eccentric phase to the concentric phase. In addition, there are similar concerns because jumping exercises using the device in Figure 1 are quite different from normal jumping exercises. If this is the case, I think it could lead to erroneous views in research on stretch-shortening cycle exercise. Throughout this manuscript, the additional loading of the eccentric phase is discussed in connection with the increase in elastic energy. However, this argument is disconcerting because the two are not necessarily the same.

Response:

- **Review Comment:** “Subjects were not giving their all due to the sudden load removal when switching from the eccentric phase to the concentric phase.”

We understand the concern, however in our protocol we had familiarisation sessions and provided feedback to participants in all jumps to ensure they were ‘giving their all’. This is supported by our EMG data which showed no significant differences in average peak or average integrated EMG between different conditions in almost all muscles (please see S4-S5 Figs, and Line 380-387) As such we do not believe that participants gave less effort in the AEL conditions compared to the body weight only conditions.

This is further evidenced in the VL EMG averaged time series data across the entire trial for all conditions (please see S6 Fig), which shows no evidence of a reduction in EMG level during the downward phase (in fact it is slightly increased with AEL conditions, although we have not statistically tested this), nor any changes in patterns due to the release in weight. Due to the rapid upward movement, it is unlikely that muscles can deactivate sufficiently quickly to change the performance, and there is no evidence in any of our EMG data that this occurs.

We have added a section to the discussion to address this concern –

L519-526: *“It is possible that untrained jumpers are not able to adapt to changes in mass as quickly as trained jumpers and could have performed submaximally in the AEL conditions due to the novelty of the task. However, care was taken to ensure jumpers had adequate training and familiarisation to the task, feedback on performance was given to try to attain maximal jump height, and we saw no decrements in activations across AEL conditions* *(see S4-S6 Figs). We expect that activation should be close to maximal for this type of task and hence an absence of EMG reductions at the time of release suggest that participants were not submaximal in the AEL conditions.”*

**Supplementary Figure 6.** Average time varying normalised VL EMG during the entire jump across conditions. The x-axis shows the average time (seconds), and the y-axis shows the normalised VL EMG. Each colour coded line represents the average value across all participants for each condition. Time 0 represents the bottom of the countermovement (turning point).

- **Review Comment:** “In addition, there are similar concerns because jumping exercises using the device in Figure 1 are quite different from normal jumping exercises. If this is the case, I think it could lead to erroneous views in research on stretch-shortening cycle exercise.”

While we accept that the constrained sled jumping used in not the same as a natural countermovement jump, we contend that this was entirely the point of the work - which aimed to isolate a potential mechanism for AEL rather than replicate prior work on the existence of AEL in jumping. That said, stretch-shortening cycle exercises exist in the form of movements as simple as a single-joint task (i.e., quick, cyclical elbow SSC curl) to movements as complicated as a countermovement jump. A jump is therefore a specific example of the general case of SSC’s, rather than vice-versa, and a more isolated SSC motion provides an opportunity to study the mechanisms underpinning that SSC’s more generally. Thus, using a modified movement task does not invalidate the scientific finding for the task in our study, particularly if our major focus was to examine whether increased tendon elastic energy storage and return could be observed in our SSC task. This is outlined in the introduction (L62-64) and then revisited in the Discussion (L407-411).

- **Review Comment:** “Throughout this manuscript, the additional loading of the eccentric phase is discussed in connection with the increase in elastic energy. However, this argument is disconcerting because the two are not necessarily the same.”

We understand that force and elastic energy are not the same - and we have been careful not to equate them in the manuscript - but they are intrinsically linked because energy stored in elastic tissues is proportional to the force applied. When the force is higher at the ground or at a joint, this implies greater energy storage in elastic tissues. We have made this clear in the introduction (L43-46) -

*“For instance, AEL CMJ may result in greater force generation in the descent to decelerate the added mass or resist the added force, potentially resulting in greater tendon loading and strain energy storage prior to the upward motion (strain of elastic tissues is directly related to force applied).”*

We have also further clarified this in the discussion –

L454-482: *“Our results refute the assumption that AEL performance effects can be attributed to enhanced ability to store and return energy with additional loading in the eccentric phase. In this study, squat depth and trunk rotation were purposefully constrained to examine differences in force output in a strictly controlled environment. Whilst the amount of mechanical work absorbed across all joints increased with increasing AEL weight, and VGRF was higher throughout most of the downward movement with increasing AEL weight (Fig 3A), there was no change in VGRF at the time of release of the weights in the AEL conditions compared to the body weight only condition. We also found no change in the ankle moment at the bottom of the movement across conditions, whilst knee joint moment increased with added weight (10.5% and 8.2% increase relative to the BW_pre_ condition in the 20% and 30% AEL conditions respectively) and the hip joint moment decreased by as much as 29% in the 30% AEL condition. Joint moments are indicative of muscle forces around joints, and therefore are directly related to energy stored in the in-series elastic tissues. Therefore the joint moment changes at the turning point of the jump with AEL suggests no change in elastic energy storage at the ankle (a key joint for storing and returning energy from the highly compliant Achilles tendon (22)), a potential small increase in energy storage across the knee, and a reduction in energy storage potential across the hip (which likely has the lowest contribution of elastic energy storage and return). Whilst the knee had some increase in potential for storage of energy with added weight (considerably less than the increase in added load relative to body weight), this did not translate into greater power or work generation at the knee. We believe this is strong evidence for limited additional elastic energy storage and return capacity with AEL, since the added weight itself did not increase the VGRF at the time of release of the added weight, nor have substantial impact on increasing joint moments. However, since our study limited the hip and trunk motion, this may have reduced the capacity for additional loading at the knee and ankle, whilst also reducing the capacity for the hip to generate force and power that might be possible in unconstrained jumping. As such, a similar mechanical analysis to that performed here is required to gauge such effects in unconstrained jumping, as previous studies have not examined this movement with such metrics.”*

Specific Comments:
Introduction

Line 42-44
In ref 9, the effects of AEL have not been investigated. This study was a cross-sectional comparison of tendon characteristics of athletes in different disciplines.

Responses:

Thank you for your comment. We have now changed the reference to another paper that suggests elastic energy return improves AEL performance.

Line 62-64
As with the comment above, I disagree with this statement.

Responses:

It is not clear exactly which part of this statement the reviewer is disagreeing with based on the previous comments. However, as stated above, we contend that our experiment involves a stretch-shorten cycle with added load in the stretch. The fact the body’s movement is constrained (or at least the trunk movement) does not invalidate this, and in fact our design was such that we remove other factors that might also contribute to enhanced performance with AEL. As stated above, we have tried to make the introduction and discussion clearer.

Reviewer #2: While seemingly simple, the execution of a countermovement jump is a complex sequence of generation and release of elastic energy. The current study is designed to determine if more elastic energy is released during the propulsive phase in response to enhancing only the eccentric load during the countermovement while controlling for all other variables. I commend the authors for designing an apparatus and methodology for controlling the many potentially confounding variables in the eccentric phase. However, this study may have been too constrained with too narrow a focus. For a complete understanding of the effects of AEL on jump performance, the entire force profile of the countermovement jump (i.e. both phases) should be examined. The study by McHugh et al. (Transl Sport Med, 2021) highlights the importance of 1) unweighting during the eccentric phase and 2) the timing of peak force to occur at the turning point, to jump performance and efficiency. While potential sources of variation such as trunk angle, and countermovement depth were controlled for in the current study, an explanation of the current findings may be found by examining eccentric phase metrics, instead of focusing only on the concentric phase. I recommend major revisions to this manuscript and the inclusion and comparison of eccentric phase force and joint metrics (see McHugh et al.) under the AEL conditions in order to completely characterize the effects of AEL on the whole countermovement jump profile.
Please see all individual comments below.

INTRODUCTION
The first paragraph of the introduction is well-written and succinctly summarizes the current research findings regrading AEL.

Lines 44-49: The amount of unweighting (defined by McHugh et al. as "low force") also plays an integral role in elastic energy storage and jump performance. While more force may be needed to decelerate during the eccentric braking phase under the AEL conditions, the amount of unweighting represents the "launch pad" from which to build tension and store elastic energy. With greater the unweighting (i.e. the lower the low force), a greater amount of tension can be accumulated and ultimately released. It is possible that AEL disrupts the mechanism of optimal unweighting, which may ultimately decrease performance. This is why the whole force curve must be examined, not only concentric phase metrics.

Responses:

Thank you for your valuable feedback and valid points about the importance of the eccentric phase in determining jump performance. We agree the force applied at the turning point depends on what occurs during the downward phase, but would like to reiterate comments made in responses to reviewer 1 that the active force is the primary factor that dictates elastic energy stored in the downward phase (force is directly related to stretch of elastic, tendinous tissues and hence energy stored). Energy stored in elastic tissues is not directly related to the work absorbed (negative work) during the downward movement, because some of this can be absorbed by muscle (ultimately output as heat). By design, energy absorbed (negative work) during the downward movement will be greater with added weight in the AEL conditions, because the gravitational force is higher and the displacement is the same. Our findings are important because the results clearly show that despite the increase in energy absorbed, there is no change in the force (either vertical ground reaction force or joint moments) at the time just before energy is returned, which means that energy stored due to the added weights is likely no different. Of course, as outlined in the discussion, this may be a function of the constraints placed on the movement, but the added mass alone is insufficient to increase the force at turning point when mass is released in the AEL conditions compared to the non-AEL condition.

We have reworded a key paragraph in the discussion to make this clearer–

L454-482: *“Our results refute the assumption that AEL performance effects can be attributed to enhanced ability to store and return energy with additional loading in the eccentric phase. In this study, squat depth and trunk rotation were purposefully constrained to examine differences in force output in a strictly controlled environment. Whilst the amount of mechanical work absorbed across all joints increased with increasing AEL weight, and VGRF was higher throughout most of the downward movement with increasing AEL weight (Fig 3A), there was no change in VGRF at the time of release of the weights in the AEL conditions compared to the body weight only condition. We also found no change in the ankle moment at the bottom of the movement across conditions, whilst knee joint moment increased with added weight (10.5% and 8.2% increase relative to the BW_pre_ condition in the 20% and 30% AEL conditions respectively) and the hip joint moment decreased by as much as 29% in the 30% AEL condition. Joint moments are indicative of muscle forces around joints, and therefore are directly related to energy stored in the in-series elastic tissues. Therefore the joint moment changes at the turning point of the jump with AEL suggests no change in elastic energy storage at the ankle (a key joint for storing and returning energy from the highly compliant Achilles tendon (22)), a potential small increase in energy storage across the knee, and a reduction in energy storage potential across the hip (which likely has the lowest contribution of elastic energy storage and return). Whilst the knee had some increase in potential for storage of energy with added weight (considerably less than the increase in added load relative to body weight), this did not translate into greater power or work generation at the knee. We believe this is strong evidence for limited additional elastic energy storage and return capacity with AEL, since the added weight itself did not increase the VGRF at the time of release of the added weight, nor have substantial impact on increasing joint moments. However, since our study limited the hip and trunk motion, this may have reduced the capacity for additional loading at the knee and ankle, whilst also reducing the capacity for the hip to generate force and power that might be possible in unconstrained jumping. As such, a similar mechanical analysis to that performed here is required to gauge such effects in unconstrained jumping, as previous studies have not examined this movement with such metrics.”*

We agree that the AEL likely disrupts the unweighting, with a different force profile required to first decelerate the combined masses and then accelerate upwards, however we are not 100% sure what metrics in the downward phase would be best examined to understand why AEL does not enhance performance in our setup. By definition, the VGRF (VGRF=ma+mg) must be higher throughout due to the added mass. As stated, work absorbed must also be higher if we have the same squat depth. There are likely some subtle differences in the acceleration profiles, due to the longer movement times (see responses to reviewer 3 below), however this may be difficult to avoid these because humans cannot instantaneously activate muscles to generate force and hence the acceleration profile is very much dictated by the constraint of the squat depth and the capacity to activate muscle. It seems that the limit to activating muscle and therefore maintaining higher force throughout the downward phase (and therefore elastic energy storage) is a major factor in preventing added performance in our AEL conditions. We already discuss the potential effects of changing descent speed (L436-446) and have made some slight modifications to a paragraph comparing results to our simulation study and activation effects –

L484-496: *“The results of this study support our earlier simulation study which found no performance enhancement in AEL for a simplified single-joint model (23). Our present study showed that added mass did not increase the VGRF at the bottom of the countermovement despite a greater mean force during the descent (Fig 3A). Mechanically, this was achieved by generating force earlier in the descent and increasing the duration of the descent (Fig 3A), which inevitably slowed down the average descent speed in AEL conditions (Fig 2C). This movement characteristic was also predicted in our simulation study (23). Our findings suggest that increased tendon-loading and elastic energy return is unlikely to contribute to the change in performance in a constrained human jumping motion, particularly when muscles are maximally activated at the bottom of the movement and producing a maximal achievable force. It is likely that other potential mechanisms, such as increasing squat depth, rate of descent or altered kinematics might be more relevant and contribute to enhanced performance.”*

METHODS
The Methods are very detailed and precisely describe each aspect of the experiment and the equipment and set-up involved.
Line 195: Joint ranges of motion and a joint stiffness measurement may be more useful to characterize any changes occurring during the eccentric phase. Also, eccentric work should be considered as potential factor as there may be an optimal amount of energy which can be absorbed during the descent before an impairment in concentric energy generation occurs.

Responses:

Thank you for your suggestion. We have considered carefully, but we are not sure this adds value here. In our study design, joint ROM was tightly controlled by the equipment constraint and the task’s kinematic constraint (i.e., same squat depth, same foot position). Therefore, in our study, the joint ROM was a “controlled variable” that should remain relatively unchanged throughout the test. Both joint stiffness (which is variable, but predictable when the joint ROM is reasonably consistent) and joint work absorbed during the descent must be greater with increasing added AEL weight. However, as stated previously, because we controlled squat depth in this study, eccentric work done on the COM (i.e., change in gravitational potential energy of BW + load mass) was simply describing different loading conditions. The problem is that this negative work cannot be attributed to energy stored elastically, as much of this can be absorbed by muscle (as heat).

However, we agree that eccentric/negative joint-level work analysis is insightful and has been completed in this manuscript re-write.

L219-220: *“We also integrated negative joint power during the eccentric phase (descent) to calculate eccentric joint work.”* This sentence was added to our Methods section.

L346-355: “*We also showed the change in negative joint work during the eccentric phase across different conditions (S3 Fig). We examined the negative work done at each joint and found significant main effects of AEL condition on negative work done at hip (p = 0.0003, η^2^_p_ = 0.086), knee (p = 0.002, η^2^_p_ = 0.143), and ankle (p = 0.033, η^2^_p_ = 0.056) joints. Overall, all three AEL conditions significantly increased the amount of negative work at the knee joint (10%: p = 0.002, d = 0.621; 20%: p = 0.001, d = 1.052; 30%: p = 0.020, d = 1.029), but significantly reduced the amount of negative work at the hip joint (10%: p = 0.001, d = 0.613; 20%: p = 0.006, d = 0.599; 30%: p = 0.004, d = 0.773). Multiple comparisons also showed that 30% AEL condition significantly increased the amount of negative work at the ankle joint compared to BW_pre_ condition (p = 0.038, d = 0.602).*”

**Supplementary Figure 3.** Box and Whisker plots for right hip, knee, and ankle joints eccentric/negative work across non-AEL (BW_pre_), 10%, 20%, and 30% AEL conditions. The x-axis shows different loading conditions, and the y-axis shows the body mass normalised joint work (J/kg). The asterisks represent a statistically significant difference from the mean of the baseline (BW_pre_) condition.

To answer the concern about optimal loading, without any information on what is likely to an optimal amount of energy to absorb – or how this influences the amount of energy absorbed vs energy elastically stored – then it is difficult to know if the added weight impairs or not. However, we hope that the inclusion of the data might be useful for people to understand what does change during the eccentric phase with added weight in the AEL conditions.

Lines 190-191: Why was average descent speed as a metric instead of maximal descent speed? The body must be decelerated from its maximum downward velocity by the end of the countermovement. The amount of force required to do this relative to the amount of unweighting represents the total amount of tension accumulated in the lower extremity. The average velocity throughout the whole countermovement does not accurately characterize the kinematics of the descent.

Responses:

Thank you for your suggestion, we’ve included maximal descent speed for our analysis. We did this by using the instantaneous velocity of the MAN marker in the eccentric phase, and the maximal descent speed was simply the scalar of this velocity. We found that all three AEL conditions had a significantly slower maximal descent speed compared to the BW condition. In this regard, whether we reported average or maximal descent speed did not influence our understanding of AEL’s impact on eccentric phase movement (i.e., which was to slow down the descent). However, to address your concern, we are happy to change the graph to maximal descent speed in Fig 2C. We also agree that maximal descent speed was mechanically important to reflect eccentric movement characteristic. Please see changes made in Line 209-210, and Line 253-263.

RESULTS
As stated earlier, the eccentric phase metric should be included in the analysis for complete characterization of the effects of AEL.

Figure 2C: I feel that maximum descent speed should be examined instead of average

Responses:

Thank you for your suggestion, we’ve changed the graph in Fig 2C.

Figure 3A: Based on the limited data presented in the graph, it does seem like there is a difference in the amount of unweighting between some of the conditions. It is difficult to determine this with certainty because the eccentric portion of the force profiles are not included. Please include the entire profile for each condition.

Responses:

Thank you for your suggestion, we’ve included the complete eccentric phase for clarity in Fig 3A. Our previous submission had a missing initial dip because we defined the start of the squat using when MAN marker dropped by 1 cm. We’ve now changed the definition of the start of the jump to *“The start of the jump was defined as the instant when the right vertical GRF decreased by more than 5 N from the initial standing phase for each trial”* (please see Line 203-205). This way, the complete eccentric phase can be included in our analysis. From the complete curves, the amount of unweighting (in terms of relative change from start to low force) did not seem to vary between conditions. Most importantly, as stated earlier, regardless of how our participants coordinated their eccentric movement strategy, our data found that it **did not affect** VGRF at the turning point in this study.

**Review Comment:** Also, it is clear that the peak concentric force does to coincide with the turning point in any of these conditions. According to McHugh et al., this is an indication of a biomechanically inefficient jump. This might be due to the constraining of so many of the eccentric phase variables using the sled. This point should be addressed in the discussion.

Responses:

Thank you for raising this point. First, we believe it is unfair to compare a constrained jump's biomechanics to an unconstrained jump's ideal characteristics. An alternative view is that our VGRF profile already showed the most efficient way to jump within the given constraints. However, whether participants used the “ideal” biomechanical strategy wasn't crucial for our study. Our goal was to see if more elastic energy was stored during the eccentric phase. As long as participants had similar kinematics (as designed in this study), we believe we eliminated the chance that they significantly changed their movements to gain an advantage, apart from the mechanical loading at the muscle-tendon level. Our results also suggest that the potential AEL effect is likely due to altering movement coordination when natural jumping is allowed, but this requires further investigation.

Figure 4A: Based on the profiles of the joint powers, it seems that there may be differences in the peak magnitude of eccentric power at the hip across conditions. However, this needs to be explored more rigorously. Additionally, the duration of the eccentric phase seems to increase in the AEL conditions, which may have implications on negative work done at each joint.

Responses:

Thank you for your suggestions. We have completed a more detailed analysis on Fig 4A and added to the below paragraphs to the manuscript:

Line 319-324: *“We also found that there was a significant main effect of AEL condition on the peak magnitude of normalised eccentric (negative) hip joint power (p = 0.002, η^2^_p_ = 0.171). There was a significant decrease in the peak magnitude of normalised eccentric hip joint power in all three AEL conditions (10%: p = 0.026, d = 0.776; 20%: p = 0.009, d = 0.823; 30%: p = 0.009, d = 0.857) compared to the baseline (BW_pre_) from the multiple comparisons.”*

Line 346-355: *“We also showed the change in negative joint work during the eccentric phase across different conditions (S3 Fig). ...Multiple comparisons also showed that 30% AEL condition significantly increased the amount of negative work at the ankle joint compared to BW_pre_ condition (p = 0.038, d = 0.602).”*

DISCUSSION
Line 322: Storage and return of elastic energy are dependent on the amount of unweighting and the total force generated during braking. As these variables were not included in the analysis, it is difficult to determine if changes to these variables were responsible for the negative effects on performance.

Responses:

This has been addressed above. We reported the energy absorbed during the eccentric phase and the timing of the movement. Whilst we acknowledge that unweighting is important for jump performance, the forces at point of release are likely to be what ultimately changes the concentric phase – and we found limited evidence of increased forces at the turning point (either VGRF or at individual joints) when the weight was released and no changes in subsequent positive work done.

Reviewer #3: SUMMARY:

The study investigates the effect of accentuated eccentric loading or AEL, on the performance of human participants executing a countermovement jump. The authors used a sliding rail mechanism to control several aspects of the jumping movement in order to reduce extraneous factors not purely related to the added load during eccentric movements. Force plates, motion capture, and electromyography were used to measure ground reaction forces, joint and center of mass kinematics, and electrical activity of notable knee extensor muscles, respectively. Linear mixed-effects models were used to estimate the effect of eccentric load (10, 20 and 30% body weight) relative to a baseline jump with minimal eccentric load on multiple parameters including jump height, descent velocity, ground reaction forces, joint moments, power, and work. The study found a slight reduction in jumping height at higher eccentric loads, perhaps due to reduced hip extensor moments and power. The authors conclude that added eccentric load does not enhance jumping performance in a controlled jumping movement.

The study is rigorously designed and executed, and the manuscript is clearly written. In particular, I appreciate the authors’ honesty in rejecting both hypotheses which are reasonably developed based on previous research on the topic. In general, I believe this paper will add a nice contribution to the literature and will point researchers toward future questions more tightly aimed at understanding the AEL phenomenon and the factors underlying it. The following are a few minor-to-moderate comments which I hope can still improve the paper further.


REVIEWER COMMENTS:

Probably my biggest comment is related to the loading strategy used in the experiment. The authors suspend weight plates from a cable attached to the backrest where participants perform the jumping movement (either 10, 20 or 30% body weight). While this approach is mostly fine, there are some limitations to consider.

First, the static loading conditions are not actually static. That is, the tension force experienced by participants is closer to the load weight plus the inertial force of the load. If we assume that tension force is always greater than zero (see next point) and the cable is sufficiently rigid, then we can also assume that the vertical motion of the load and sliding rail are roughly equivalent during experiments. As such, any accelerations of the participants in the vertical direction also contribute to fluctuations in tension force. This could be one reason why descent speeds were slower at greater loads; that is, participants may have been avoiding greater tension fluctuations during descent by employing lower accelerations to reach lower descent speeds.

Responses:

As the reviewer indicates, forces experienced by the body are due to body weight + AEL weight and due to acceleration of the body mass and additional AEL mass. Because the accelerations experienced by the body and additional mass should be equivalent, then the force experienced should be the same through the cable as it would be if the weight was being held directly on the body. As such, the forces experienced by the body (i.e., VGRF) is a direct measure of the total force required to accelerate the body and added mass and there is not a requirement to consider the mass separately. We stress that our aim was not to apply a constant force.

We have addressed this concern in more detail in response to the specific comments below.

Second, if the participant ever accelerates downward at a rate higher than gravitational acceleration, then the tension in the cable can drop to zero. It is theoretically possible that individuals may have slowed their descent rate at loads greater than zero in order to prevent a zero tension eventually resulting in a large force spike once the weight caught tension again. This scenario is perhaps unlikely for multiple reasons, but since there is no measurement of tension force or acceleration during the eccentric motion, the reader cannot know this for sure.

Responses:

As stated above, the vertical ground reaction force is indicative of the accelerations experience by the body and the added mass. The downward acceleration of the mass during descent (equivalent to that of the body if we consider the cable relatively inextensible) does mean the tension in the cable drops, alongside that of the force on the body, however VGRF force never approaches forces that are close to zero, and as such the tension in the cable must always be positive and the cable was always taut. Visual inspection showed this to be the case. We have further addressed this below.

Third, another form of tension fluctuation could have occurred due to centripetal accelerations of the weight plates swinging away from a vertical orientation. While the motion of the participants was fairly well-controlled, it is unclear whether the researchers made any attempt to control the motions of the weight plates during descent. This would perhaps have only a small effect, however.

Response:

We have added more details on any potential fluctuations in force due to movement of the mass, which we tightly controlled.
Line 135-138: “*We ensured that the weight plates were static prior to the start of the trial, which ensured that the downward movement of the mass did not incur additional horizontal movements that might contribute to added forces in the cable through centripetal accelerations.*”

In each of the above points, questions about the load being applied arise due to a missing measurement of the tension force during the experiment. While it would likely not be realistic to redo experiments with these measurements, a fairly accurate approximation of the load force could be calculated via the summation of inertial force and load weight: Tension=mg+my ̈ where y ̈ is vertical acceleration of the load (equal to vertical acceleration of the participant) and m is the load mass. Average tension during descent could be measured and even included in the statistical model as continuous data (rather than categorical) in order to potentially account for more of the data’s variability with loading condition. At the very least, it would be helpful to show an example of approximated tension from the calculation somewhere in the manuscript.

Response:

We agree that the Tension=m_w_g+m_w_y ̈, where m_w_ is the mass of the added weight [m_w_ = 0.1m_b_, 0.2m_b_, or 0.3m_b_]. If we assume that the acceleration of the body is the same as that of the mass (they move in the same direction in our setup) due to the wire being relatively inextensible, then VGRF = (m_b_+m_w_).g + (m_b_+m_w_). y ̈; [m_b_ is the body mass]. If we rearrange the formula, VGRF = m_b_(g + y ̈) + m_w_(g + y ̈). The contribution of m_w_ is just a proportion of the VGRF based on the ratio of m_w_ to m_b_. For the cable to be slack then the force due to acceleration would have to cancel that of the force due to gravity. Looking at our force profiles, the accelerations are not sufficient for this to be close to a reality. Furthermore, VGRF can never be negative in our setup (i.e., there’s no way to pull or push the body down) and therefore the downward acceleration of the person can never exceed 1g, which is the only way of slackening the cable.

We want to make it clear that our intention was not to apply a constant force, but to apply an inertial force similar to that used in practice. The tension will change across added mass conditions (tension will be greater for greater mass as the acceleration profiles are rather similar in this constrained task), but this is no different to other AEL experiments (e.g., handheld) where the mass also accelerates with the body. As such, we do not think that fluctuations in cable tension is a large factor. We have addressed this in the discussion.

Line 527-529: *“Fluctuations in the tension in the cable with acceleration and deceleration of the added mass could have influenced the chosen motor pattern, however the accelerations were insufficient to cause significant reductions in force applied."*

In terms of the average tension during descent, this value should be equal to the static load weight (m_w_ g) because the average acceleration in the descent should be zero (both starting and ending at zero velocity), regardless of how you move down. Therefore, we do not think that including calculation of the average tension provides any additional information to our study.

Line 119 – was friction in the linear rail system or pulleys assessed? Can the authors estimate how much additional load any friction or neglected inertial effects might have added? If not, it might be more transparent to mention that these effects were neglected in the analysis but are assumed to be quite low.

Response:

We did not assess the pulley friction and hence cannot estimate the effects of adding weight, however we assume that it is near negligible in comparison to considerable extra force due to the weight. We added this assumption to the methods.

Line 126-127: *“The friction in the linear rail system and pulleys was not considered in our analysis but was assumed to be negligible.”*

Line 122 – when the author refer to an “inextensible” metal wire, maybe they can report the gauge or diameter of the wire to help back up their claim of rigidity.

Response:

We’ve added this information on Line 125, thank you: *“(diameter: 5 mm, marine grade stainless steel cable)”*

Lines 144, 177, 198 - in most cases, a number value is reported followed by a space and the units. However, there are a few instances where there is no space between the value and the units. Some examples are “9mm” (Line 144), “15Hz” (Line 177) and “5Hz” (Line 198). The authors should check the manuscript matches the journal formatting rules.

Responses:

We’ve made changes as requested, thank you.

Line 128 – is it possible that the noise of the weights hitting the box could have affected participant performance? Perhaps this is part of a slightly larger discussion regarding participant instructions. For example, were participants told about what would happen to the weights during each jump? Also, how was “maximal effort” (Line 100) enforced or motivated?

Response:

Participants were explained the protocol and they already experienced the noise of drop weights hitting the box in the familiarisation session. They were prepared and aware of the noise, which did not come as a surprise to them. In our opinion, the noise of drop weights hitting the box was unlikely to impact performance outcomes in the data collection session. Maximal effort was motivated by providing verbal instructions to the participants by saying “jump as high as possible” after each maximal trial.

We have added more details in the methods as suggested -

Line 101-102: *“Participants were verbally motivated with the instruction to “jump as high as possible” after each maximal trial.”*

Line 132-135: *“Since participants were explained the protocol and were trained with the noise of drop weights hitting the box in the familiarisation session (session one), we believe that participants were aware of the noise and were unlikely to be hugely affected by the noise during jumping in the data collection (session two).”*

Line 324 – it is interesting that the previous simulation study predicted some of the experimental results, and the discussion that follows in Lines 392-398 adds a new dimension to the interpretation. However, the initial mention of the simulation study in Line 324 feels out of place since no further context is given. I recommend waiting until the later discussion before referencing the simulation.

Response:

We have now removed this sentence as suggested, thank you.

Line 395 – descent is misspelled here as “desent.”

Responses:

We’ve made change as requested, thank you.

Line 431-432 – I am not familiar with the studies referenced, but perhaps it is also possible that subjects in these studies could have pushed off the released weights in order to gain some amount of extra upward impulse.

Response: Thank you for your suggestion. We’ve included this speculation in our discussion. However, this idea remains speculative as there were no force/pressure data reported between the hands the weight. Please see Line 536-539: “*It was also possible that participants in these studies (2, 4) could have pushed off the released weights in order to gain extra upward impulse to further enhance jump performance. However, no data were available to confirm this speculation.*”

The manuscript could be improved by adding either some discussion or background regarding the impact of the study and the topic more generally. Beyond developing a better understanding of the specific phenomenon related to human jumping, do the findings (or lack thereof) tell us something useful about training strategies or sports performance, for example?

Response:

We have the added following section at the end of the discussion.

Line 541-549: *“Our study provides an alternative view on how AEL might impact human lower limb work and power production during explosive movements. While AEL involves decelerating a heavier load in the eccentric phase, how the motion was performed was likely more critical than simply using a higher load mass. A higher elastic energy storage could only be achieved by a higher muscle force at the start of the push-off, whereas our study showed this did not occur in a constrained version of AEL. Our study provides evidence against a benefit of AEL for other similar movement configurations, such as for use in knee press machines or knee extension sleds. However, more research is required to inform coaching decision related to AEL implementation during natural, unconstrained jumps.”*
