# Supplementary material for: Increased force and elastic energy storage are not the mechanisms that improve jump performance with accentuated eccentric loading during a constrained vertical jump
Source: PLoS One. 2024 Aug 6;19(8):e0308226. doi: 10.1371/journal.pone.0308226 (PMC11302863; doi:10.1371/journal.pone.0308226)
Supplement: Attachment — Submitted filename: Response to Reviewers.docx [file pone.0308226.s009.docx]

Reviewer #1: Thank you for your responses. As pointed out previously, however, I disagreed with two points.
Firstly, the reduction of the works of hip joint and center of mass with higher eccentric loading conditions may be related to the fact that the participants were not giving their all due to the sudden load removal when switching from the eccentric phase to the concentric phase. The authors rejected this possibility, because no differences in EMG data among all conditions were found. According to the individual EMG data (Figure 6), it is recognized that there were significant individual differences in the changes in EMG data in response to loading conditions. The individual differences were particularly pronounced in the GLUT (Fig. S4) and BF (Fig. S5). Because these muscle groups were closely related to hip joint work, I do not consider it possible to fully explain why the points I have identified (see above) are not involved in the results of this study (lower hip work with heavy loading).
Secondly, the additional loading of the eccentric phase is discussed in connection with the increase in elastic energy in this study. However, this argument is disconcerting because the two are not necessarily the same. Previous studies showed that optimal loading of the ECC phase existed with squat exercise (Takarada et al. 1997), drop-jump (Komi and Bosco 1978). For example, Takarada et al (1997) reported that power output during the concentric phase increased initially with the eccentric force, whereas they began to decline when the eccentric force exceeded >1.4 times the sum of load and body weight (JAP 83: 1749-1755).

Response:

Concern with submaximal effort with AEL -

After carefully reviewing EMG data in Figure 6, Figure S4 and Figure S5, we agree that our EMG data showed some inter-individual variability in the EMG responses in muscles across the hip joint (GLUT and BF). However, single muscle EMG response can be highly variable across individuals for multi-joint dynamic movements (i.e., jumps), and seeing individual variability in EMG response does not necessarily mean submaximal voluntary effort in the jumps.

We have acknowledged the possibility that participants may not use maximal effort during AEL jumps in the discussion of the manuscript, but this is hard to test in our study based on the available EMG data. As our EMG data did not show any statistically significant difference between conditions for GLUT and BF muscles, our overall data do not generally support this assertion. We have addressed this concern in the manuscript (L522-L527).

L522-L527: “However, care was taken to ensure jumpers had adequate training and familiarisation to the task, feedback on performance was given to try attaining maximal jump height, and we saw no statistical decrements in activations across AEL conditions (see S4-S6 Figs). We did observe inter-individual variability in the EMG response to different conditions (S4-S5 Figs), and therefore we cannot rule out that some of our analysed jumps did not achieve maximum activation within the constraints of the movement task.”

L527-L529: We removed this sentence in our manuscript -“We expect that activation should be close to maximal for this type of task and hence no reductions at the timing of release suggest that participants were not submaximal in the AEL conditions.”

Concern with “additional loading of the eccentric phase is discussed in connection with the increase in elastic energy in this study” -

We believe that the reviewer is making the argument that elastic energy storage is related to the absorption of energy during the eccentric phase, and not to the load experienced and that as such we ‘should’ find that energy return is higher unless our design or constraints prevent maximum effort. The reviewer mentioned two studies (Takarada et al. 1997; Komi and Bosco 1978) using a range of loads to determine “optimal load” for the movements being tested, but these studies assessed power output with different loads in the concentric phase. Whilst we understand the reviewer’s point of view, we think there are a number of differences between our study and those cited, which make the comparison unfair.

The cited studies (Takarada et al. 1997; Komi and Bosco 1978) used a **fixed load** during uncontrolled jumps - these were non-AEL tasks. The typical power-load relationship during squat exercise (Takarada et al. 1997) was the combined effect of both the concentric muscle force and the concentric movement velocity, with the latter being heavily constrained by the additional load in the body-mass system when the load mass exceeded a certain limit (1.4 times body + load mass); higher mass slows the concentric phase allowing muscles to generate more power according to the velocity-power relationship. However, this well-established finding cannot explain our AEL jump study because our concentric load was always the same load (relatively light load), regardless of the conditions. In our experiment, we also ensured we controlled squat depth (with the velocity of the squat also being matched, although not specifically controlled). Whilst we see from the Takarada et al. (1997) study that there was an increase in power with weight up to 1.4 times body + load mass, and that this was related (although not statistically tested) to a small increase in force prior to the upward movement, this experiment did not control squat depth. Similarly, the Komi and Bosco (1978) study also did not control squat depth and the input energy from changing drop jump height cannot be considered equivalent to our study. Furthermore, the relationship between negative work and elastic energy is not straight forward, given that ultrasound studies have clearly shown that muscles (particularly at the knee) actively absorb some of the energy by lengthening rather than storing all of this energy as was assumed in the Komi and Bosco (1978) paper (see for example Nikolaidou et al, Royal Society Open Science, 2017).

When we calculate the mechanical energy absorbed in the eccentric phase, we see that this increases with increased load; this must be the case as we controlled squat depth (greater mass, same displacement = great change in potential energy). However, this is clearly not related to increased jump height in our results as we do not see any increase in jump height. The primary aim of our study was to objectively assess whether the VGRF at the turning point was also higher with AEL, given a higher mean VGRF during the eccentric phase. We then used this data to evaluate whether this relates to greater power/work in the upwards phase. We believe we have made a sound argument to link the forces at the instant at the turning point to potential elastic energy storage (L43-46) and justify these interpretations further in detail in the discussion, with the added use of the word ‘We believe’ to ensure the reader understands that the interpretation is our own and not necessarily proven (see L454-L482):

*“We believe our results refute the assumption that AEL performance effects can be attributed to enhanced ability to store and return energy with additional loading in the eccentric phase. In this study, squat depth and trunk rotation were purposefully constrained to examine differences in force output in a strictly controlled environment. Whilst the amount of mechanical work absorbed across all joints increased with increasing AEL weight, and VGRF was higher throughout most of the downward movement with increasing AEL weight (Fig 3A), there was no change in VGRF at the time of release of the weights in the AEL conditions compared to the body weight only condition. We also found no change in the ankle moment at the bottom of the movement across conditions, whilst knee joint moment increased with added weight (10.5% and 8.2% increase relative to the BWpre condition in the 20% and 30% AEL conditions respectively) and the hip joint moment decreased in by as much as 29% in the 30% AEL condition. Joint moments are indicative of muscle forces around joints, and therefore are directly related to energy stored in the in-series elastic tissues. Therefore the joint moment changes at the turning point of the jump with AEL suggests no change in elastic energy storage at the ankle (a key joint for storing and returning energy from the highly compliant Achilles tendon (22)), a potential small increase in energy storage across the knee, and a reduction in energy storage potential across the hip (which likely has the lowest contribution of elastic energy storage and return). Whilst the knee had some increase in potential for storage of energy with added weight (considerably less than the increase in added load relative to body weight), this did not translate into greater power or work generation at the knee. We believe this is strong evidence for limited additional elastic energy storage and return capacity with AEL, since the added weight itself did not increase the VGRF at the time of release of the added weight, nor have substantial impact on increasing joint moments. However, since our study limited the hip and trunk motion, this may have reduced the capacity for additional loading at the knee and ankle, whilst also reducing the capacity for the hip to generate force and power that might be possible in unconstrained jumping. As such, a similar mechanical analysis to that performed here is required to gauge such effects in unconstrained jumping, as previous studies have not examined this movement with such metrics.”*

We have addressed numerous limitations or cautions in interpretations (including around maximal effort) in the discussion to ensure that the reviewer’s concerns are also considered alongside our interpretation.

Reviewer #2: The authors have thoroughly addressed my comments raised in a previous round of review. I feel that this manuscript is now acceptable for publication. Well done!

Reviewer #3: In general, I am satisfied by the author responses to the previous round of comments. I have listed a few minor editorial comments for the authors to consider, but otherwise I am happy to recommend publication.

Note, the following line numbers refer to the tracked-changes version of the manuscript.

Line 100: the way this line is written could be misunderstood by the reader to mean that the additional mass is spatially configured on top of the backrest. Perhaps this line could be updated for clarity: “…other AEL conditions included extra mass in addition to the mass of the backrest.”

Response: We have updated the sentence as requested (see L99-L100).

Line 198: The letter “s” in OpenSim should be uppercase.

Response: We have changed the letter “s” to uppercase for all OpenSim words in the manuscript that were in lowercase (see L192 and L197).

Line 483: change to “…the added weight, nor did it have…”

Response: We have updated the sentence as requested (see L476).

Line 528: the word novelty is misspelled as “novely”

Response: We have corrected the spelling as requested (see L521).

Line 535-536: it is unclear what qualifies as a “significant reduction in force applied”, since I do not believe the authors are referring to statistical significance here (if they are, the statistics should be reported, e.g., p-value, etc.). Otherwise, I suggest a slight rewording, e.g., “…however the accelerations were unlikely to have caused a substantial reduction in the force applied.” Furthermore, this statement could be strengthened by quantification, e.g., “accelerations caused an average fluctuation in the force applied of X % participant body weight, and so are unlikely to have had a large influence on the motor patterns of participants.”

Response: We have changed the sentence to “…however the accelerations were unlikely to have caused a substantial reduction in the force applied.” (L531-532).

Line 554: Configuration should be plural. Similarly, knee press machine and knee extension sled should be plural, or else they should be preceded by the word “a”, e.g., “such as for use in a knee press machine…”

Response: We have changed these words to plural (i.e., configurations, machines, sleds) in L550-L551.
